# A new challenge for expert witnesses relying on subjective information

**DOI:** 10.1080/20961790.2017.1342587

**Published:** 2017-06-28

**Authors:** Barry A J Fisher (Retired)

**Affiliations:** Crime Lab, Los Angeles County Sheriff's Department, Indio, CA, USA

Forensic Scientists increasingly face challenges when testifying about pattern evidence. “Pattern evidence” here denotes fingerprint evidence, firearms, tool marks, footwear, tire impressions, handwriting, bite marks, etc. Critics question whether experts using only subjective information can provide the court with reliable information. Detractors maintain that objective data, meaning statistics, is necessary for the expert to form an opinion in court cases. Some critics have used the depreciatory term “junk science” to suggest that certain forensic science disciplines have little or no validity and should no longer be used.

This short commentary offers a different assessment and argues for the value of pattern evidence in court cases. Suggesting that knowledge without statistics to assess its worth is a rather narrow point of view. To propose that without statistics, evidence interpretation has little probative value to assist a judge or jury in reaching a finding fails to consider how conclusions are made. It also suggests that any sort of expert opinion without objective data should not be used in court.

The question of admissibility of expert evidence goes beyond the use or even the availability of objective data to support an expert's opinion. Many experts offer their conclusions in legal matters based on their professional training, knowledge, skills and experience. Often that knowledge is based on empirical data. Consider, psychologists who offer opinions about syndrome evidence, or forensic pathologists who offer opinions on the manner and means of death. Medical practitioners base diagnoses on practical experience, which may not have much statistical data to support conclusions. Practitioners would call that professional expertise. If there were the requirement that statistics had to be provided for pattern evidence cases, then all expert testimony would be held to that same requirement. The very purpose of expert witnesses is to assist judges and juries to understand matters beyond their ken. Opinion testimony would rely solely on the availability of objective data and subjectivity would limited or perhaps not be permitted.

The defense bar, law school professors, academics and statisticians make the claim that statistical data is essential to quantify opinion testimony; that without statistics, opinion evidence should not be admitted in court to support a conclusion. While these stakeholders certainly have every right to make their claims, it is only the trial court judge who is the “gatekeeper” and who decides what is or is not admissible. That is not to say that statistics are not important, but where statistical information is limited, opinion evidence based on subjectivity ought not be dismissed out of hand. Rather the information should be presented to the court to make decisions on the weight and admissibility of the evidence.

In the United States, courts have long wrestled with the question of who is an expert and what is expert testimony. The current rules about expert testimony (at least for federal courts – State courts have variations on these) are found in the Federal Rules of Evidence (FRE) [1].

FRE Rule 702 under Testimony by Expert Witnesses states:

A witness who is qualified as an expert by knowledge, skill, experience, training, or education may testify in the form of an opinion or otherwise if:

(a) the expert's scientific, technical, or other specialized knowledge will help the trier of fact to understand the evidence or to determine a fact in issue;
(b) the testimony is based on sufficient facts or data;
(c) the testimony is the product of reliable principles and methods; and
(d) the expert has reliably applied the principles and methods to the facts of the case.

Further, FRE Rule 703 under the Bases of an Expert:

An expert may base an opinion on facts or data in the case that the expert has been made aware of or personally observed. If experts in the particular field would reasonably rely on those kinds of facts or data in forming an opinion on the subject, they need not be admissible for the opinion to be admitted. But if the facts or data would otherwise be inadmissible, the proponent of the opinion may disclose them to the jury only if their probative value in helping the jury evaluate the opinion substantially outweighs their prejudicial effect.

The *Notes of Advisory Committee on Proposed Rules* [2] go into considerable detail on interpreting how judges should apply the rules. The theme that runs through how judges interpret expert opinions is the reliability of those opinions. While the Federal Rules and court cases lay out guidelines for trial courts, such as testimony on error rates, publication of procedures, judges are given considerable leeway on how to apply the criteria.

In 2009, the National Academy of Sciences (NAS) issued it landmark report about forensic science, *Strengthening Forensic Science in the United States: A Path Forward* [3]. The report outlined a number of areas in forensic science practice, which need further discussion. It noted that some forms of forensic science were more robust than others and recommended the need for further research.

A result of the NAS report was the creation of the Organization of Scientific Area Committees (OSAC) for Forensic Science [4] or OSAC under the NIST, The National Institute of Standards and Technology, US Department of Commerce. OSAC has made progress to address some of the issues raised in the NAS report.

More recently, The President's Council of Advisors on Science and Technology (PCAST) issued a report entitled *Forensic Science in Criminal Courts:* Ensuring Scientific Validity of Feature-Comparison Methods [5].

The report focused on a number of issues. The principal attention concerned the reliability of pattern evidence. It challenged the validity of evidence where feature comparative methods are used to associate items of evidence. Examiners observe patterns found within the evidence which may associate an item of evidence to another item or a particular source. The most common types of pattern evidence are fingerprints, but other types of pattern evidence, such as firearms and tool mark evidence, tire impression and footwear evidence, handwriting evidence, bite mark evidence, were also highlighted. In addition, the report also discussed DNA cases where mixtures of biological material were found, such as in the case of multiple assailant sexual assault cases.

PCAST highlighted the importance of statistics to inform the trial judge about the significance of the evidence and by extension the degree of importance to which it should be given by a jury. It reported that in some cases, there is insufficient data to reliably make a conclusion about the connection between an item of evidence collected at a crime scene and a reference sample, for example a shoe impression in soil at a burglary scene and a suspect's shoes and points out that there is insufficient information to ascribe a statistical value to the significance of an examiner's observations.

However, the issue here is not only the presence or lack of statistics to inform judges of their gatekeeping responsibility. A footnote from the *Kumho Tire Co. v. Carmichael* case summarizes a key point:

The Daubert “gatekeeping” obligation applies not only to “scientific” testimony, but to all expert testimony. Rule 702 does not distinguish between “scientific” knowledge and “technical” or “other specialized” knowledge, but makes clear that any such knowledge might become the subject of expert testimony. It is the Rule's word “knowledge,” not the words (like “scientific”) that modify that word, that establishes a standard of evidentiary reliability. Daubert referred only to “scientific” knowledge because that was the nature of the expertise there at issue. Neither is the evidentiary rationale underlying Daubert's “gatekeeping” determination limited to “scientific” knowledge. Rules 702 and 703 grant all expert witnesses, not just “scientific” ones, testimonial latitude unavailable to other witnesses on the assumption that the expert's opinion will have a reliable basis in the knowledge and experience of his discipline. Finally, it would prove difficult, if not impossible, for judges to administer evidentiary rules under which a “gatekeeping” obligation depended upon a distinction between “scientific” knowledge and “technical” or “other specialized” knowledge, since there is no clear line dividing the one from the others and no convincing need to make such distinctions.

Certainly, objective data about any sort of expert opinion evidence is helpful, but the absent that information should not necessarily exclude the information from a jury.

The reader might well think that the information in this commentary does not really cover much new ground. However, this writer suspects that new forensic examiners as well as new prosecutors may not have considered this issue, and may get caught up in an evidentiary hearing with the goal of excluding certain physical evidence. So, it is certainly worth considering. In addition, judges often lack expertise in scientific and technical subjects and need assistance in understanding how all this fits together.

The admissibility of expert evidence has yet to be fully resolved. Likely, there will be additional court decisions that will provide direction to trial court judges and forensic experts. For now, all we can do is to wait and see how this all plays out.
